# Variance of the global signal as a pretreatment predictor of antidepressant treatment response in drug-naïve major depressive disorder

**DOI:** 10.1007/s11682-018-9845-9

**Published:** 2018-02-23

**Authors:** Jiajia Zhu, Huanhuan Cai, Yonggui Yuan, Yingying Yue, Deguo Jiang, Ce Chen, Wei Zhang, Chuanjun Zhuo, Yongqiang Yu

**Affiliations:** 1 Department of Radiology, The First Affiliated Hospital of Anhui Medical University, Hefei, China; 2 Medical Imaging Center, The First Affiliated Hospital of Anhui University of Chinese Medicine, Hefei, China; 3 Department of Psychosomatics & Psychiatry, Institute of Psychosomatics, Zhongda Hospital, Medical School of Southeast University, Nanjing, China; 4 Department of Psychiatry, Wenzhou Seventh People’s Hospital, Wenzhou, China; 5 Department of Psychiatry, Tianjin Mental Health Center, Tianjin, China

**Keywords:** Major depressive disorder, Functional magnetic resonance imaging, Global signal, Antidepressant treatment, Prediction

## Abstract

Several behavioral and neuroimaging markers could be used to predict eventual antidepressant medication (ADM) outcomes in patients with major depressive disorder (MDD). However, these predictors are either subjective or complex, which has limited their clinical use. Thus, we aimed to identify an objective and easy-to-get marker to predict early therapeutic efficacy. Forty-seven drug-naïve patients with MDD and 47 age-, gender- and education-matched healthy controls underwent resting-state functional magnetic resonance imaging (fMRI) scans. We calculated the variable coefficient (VC) of the global signal for each subject. Baseline Hamilton Rating Scale for Depression (HRSD) score and that after 2 weeks of ADM were assessed for patients. Although there was no difference in VC between patients with MDD and healthy controls, we found a significant positive correlation between the VC and the decline rate of HRSD scores in the patients. Compared with the non-responding depression (NRD) group, the treatment-responsive depression (TRD) group had a higher VC. Receiver operator characteristic curve analysis revealed that the VC exhibited a good ability to differentiate TRD from NRD. In addition, the linear and logistic regression analyses showed that the VC was a significant predictor of the decline rate of HRSD scores and the antidepressant treatment response. These findings suggest that variance of the global signal may serve as a useful marker to help clinicians find an appropriate drug for individuals with MDD at the earliest opportunity and then further to facilitate personalized therapy.

## Introduction

Major depressive disorder (MDD) is one of the most prevalent and debilitating psychiatric disorder (Wang et al. 2007). Given its significant contribution to loss of productivity, low quality of life, and suicide (Collins et al. 2011; Kupfer et al. 2012), MDD has become one of the biggest health challenges, which causes increasing social and economic burdens (Ustun et al. 2004). Currently, antidepressant medication (ADM) is the most common treatment for MDD (Marcus and Olfson 2010). However, only one-third of patients with MDD achieve remission with ADM; among these remitted patients, 50% experience relapse before they achieve recovery (Rush et al. 2006). The ADM treatment development has lagged because of a lack of widely accepted biomarkers available to predict antidepressant treatment response.

Predicting a prognosis in an early phase of antidepressant treatment may facilitate an effort to find an appropriate drug for individuals with MDD at the earliest opportunity (Nakajima et al. 2010). Previous studies have demonstrated that early changes in joy, motivation, depressive symptoms, plasma catecholamine metabolites, repeated cortisol awakening response, quantitative electroencephalography biomarkers after a short time of ADM (e.g., 2 weeks) could predict eventual antidepressant treatment outcomes in depressed patients (Beck et al. 2015; Gorwood et al. 2015a, b; Hunter et al. 2010; Sakurai et al. 2013; Ueda et al. 2002; Vermeiden et al. 2015). Furthermore, evidence from neuroimaging studies suggests that interhemispheric asynchrony and disrupted network topological configurations could also serve as pretreatment predictors of early antidepressant response in MDD (Hou et al. 2016a, b). However, these predictive markers are either subjective (e.g., assessment of joy, motivation, depressive symptoms) or complex (calculation of voxel-mirrored homotopic connectivity and network topological properties), which has limited their clinical use. Thus, an objective and easy-to-get marker is needed to predict early therapeutic efficacy and then further to guide personalized therapy.

For resting-state functional magnetic resonance imaging (fMRI) data, the global signal has been thought to reflect non-neuronal noise (e.g., physiological, movement, scanner-related) (Chang and Glover 2009; Power et al. 2014, 2016). Global signal regression (GSR) has been used as a standard step during the processing of resting-state fMRI data (Macey et al. 2004). Recently, GSR has been considered a controversial topic in resting-state functional MRI analyses (Chai et al. 2012; Chen et al. 2012; Fox et al. 2009; Murphy et al. 2009; Murphy and Fox 2016; Qing et al. 2015; Saad et al. 2012) because the global signal has also been found to reflect neurobiologically important information (Power et al. 2016; Scholvinck et al. 2010). For example, schizophrenia patients exhibit increased variance in the global signal (Yang et al. 2014); caffeine can lead to a reduction in global signal amplitude (Wong et al. 2012); global signal amplitude is related to electroencephalographic (EEG) vigilance measures (Wong et al. 2013); there are differences in global signal amplitude between the eyes open and eyes closed states (Wong et al. 2016). Combined, the global signal has the potential to be a clinically relevant marker in brain disease.

In the present study, we used resting-state fMRI data to investigate the relationship between the global signal and short-term antidepressant response in patients with MDD. We aimed to identify an objective and easy-to-get marker to predict early treatment outcome of MDD. We hypothesized that depressed patients with different variance in the global signal would show different response to treatment.

## Methods

### Participants

A total of ninety-four right-handed individuals were enrolled in the present study, including 47 drug-naïve patients with MDD recruited consecutively from the psychiatric outpatient or inpatient department of the local hospital and 47 healthy controls recruited from the local community via advertisements. The patients and controls were well-matched in terms of age, sex and education (Table 1). The diagnosis of MDD was made according to the Structural Clinical Interview of the DSM-IV (SCID) (First et al. 1997), patient edition. The severity of depression was assessed using the 24-item Hamilton Rating Scale for Depression (HRSD-24) (Williams 1988). Only those patients with a HRSD-24 score ≥ 20 were eligible for this study. Healthy controls were carefully screened for a current or lifetime diagnosis of any Axis I and II disorder using the SCID, non-patient edition. Exclusion criteria for all participants were (1) the presence of other Axis I psychiatric disorders such as schizophrenia, bipolar disorder, substance-induced mood disorder, anxiety disorders, substance abuse or dependence; (2) a history of neurological diseases or other physical illness; (3) a history of head injury resulting in loss of consciousness; (4) the inability to undergo an MRI. In addition, all healthy controls reported no psychiatric disorders among their first-degree relatives. This study was approved by the local ethics committee, and written informed consent was obtained from all participants after they had been given a detailed description of the study.

**Table 1:** Demographic and clinical characteristics of the sample

Characteristics	MDD	HC	Statistics	*P* value
Number of subjects	47	47		
Age (years)	46.4 ± 13.5	47.0 ± 17.9	*t* = 0.182	0.856 ^b^
Sex (female/male)	27/20	23/24	χ^2^ = 0.684	0.408 ^c^
Education (years)	11.2 ± 3.8	11.7 ± 4.1	*t* = 0.657	0.513 ^b^
FD	0.141 ± 0.066	0.149 ± 0.073	*t* = 0.601	0.549 ^b^
HDRS_baseline_	30.3 ± 7.1	–		
HDRS_2-weeks_	15.0 ± 7.7			
Decline rate of HDRS scores	50.4% ± 22.3%			
Illness duration (months) ^a^	23.7 ± 36.1	–		
Onset age (years) ^a^	43.4 ± 12.4	–		
Episode number ^a^	1.3 ± 0.7	–		
Current episode duration (months)	5.0 ± 6.3	–		

The data are presented as the mean ± SD. *Abbreviations: FD* frame-wise displacement, *HC* healthy controls, *HDRS* Hamilton Depression Rating Scale, *MDD* major depressive disorder

^a^ The data are available for 39 of 47 patients

^b^ The *P* values were obtained by two-sample *t*-tests

^c^ The *P* value was obtained by Chi square test

The MRI scans and the baseline HRSD were completed 1 day before the patients started to receive ADM. The ADM consisted of selective serotonin reuptake inhibitor (SSRIs), serotonin-norepinephrine reuptake inhibitor (SNRIs) and an agglomeration of antidepressant combinations (SSRIs, SNRIs or mirtazapine). After 2 weeks of ADM, the patients completed the HRSD again. The decline rate of HRSD scores is defined as (HRSD_baseline_ – HRSD_2-weeks_)/HRSD_baseline_ × 100%. The detailed clinical characteristics of the patients are shown in Table 1, including the HDRS score, illness duration, onset age, episode number, and current episode duration.

### Data acquisition

MRI data were acquired using a 3.0-Tesla scanner (Magnetom Verio, Siemens, Erlangen, Germany). Tight but comfortable foam padding was used to minimize head motion, and earplugs were used to reduce scanner noise. High resolution structural images were acquired sagittally using a 3D T1-weighted magnetization-prepared rapid gradient-echo (MPRAGE) sequence with the following parameters: repetition time (TR) = 1900 ms; echo time (TE) = 2.48 ms; inversion time (TI) = 900 ms; flip angle (FA) = 9°; field of view (FOV) = 250 mm × 250 mm; matrix = 256 × 256; slice thickness = 1 mm, no gap; slice number = 176; and acquisition time = 258 s. Resting-state functional blood-oxygen-level-dependent (BOLD) images were acquired axially using a gradient-echo echo planar imaging (GRE-EPI) sequence with the following parameters: TR/TE = 2000/25 ms; FA = 90°; FOV = 240 mm × 240 mm; matrix = 64 × 64; slice thickness = 4 mm; no gap; slice number = 36; 240 volumes; and acquisition time = 480 s. Before the scanning, all subjects were instructed to keep their eyes closed, relax, move as little as possible, think of nothing in particular, and not fall asleep during the scans. During and after the scanning, we asked subjects whether they had fallen asleep to confirm that none of them had done so. All MR images were visually inspected to ensure that only images without visible artifacts were included in subsequent analyses.

### fMRI data preprocessing

**Fig. 1:**
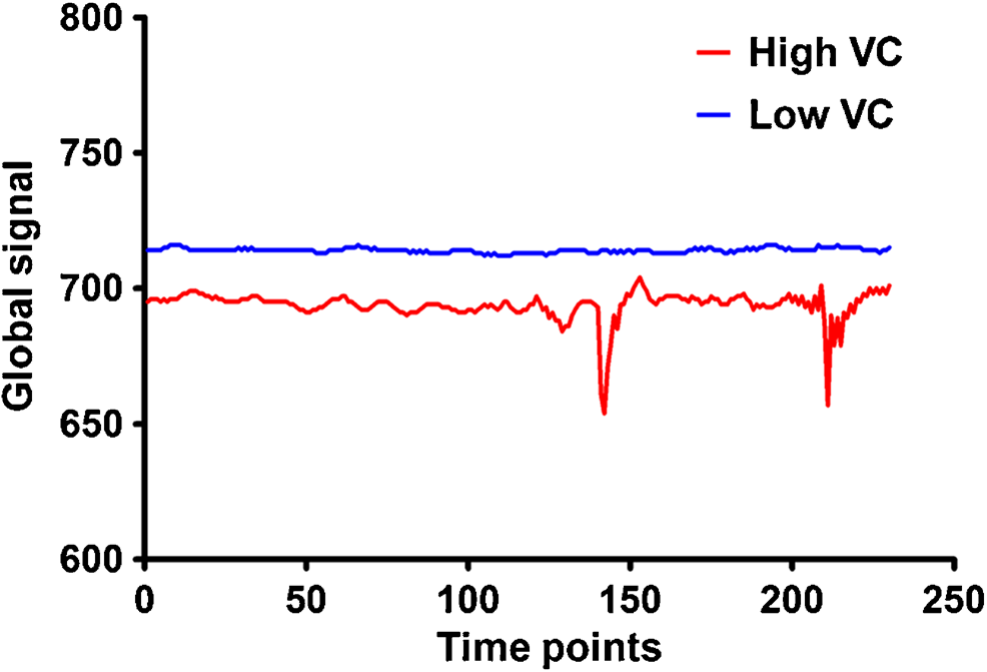
Illustration of the variable coefficients of the global signal for two individuals. Abbreviations: VC, variable coefficient

BOLD MRI data were preprocessed using SPM8 (http://www.fil.ion.ucl.ac.uk/spm). The first 10 volumes for each participant were discarded to allow the signal to reach equilibrium and the participants to adapt to the scanning noise. The remaining volumes were corrected for the acquisition time delay between slices. Then, realignment was performed to correct the motion between time points. All participants’ BOLD data were within the defined motion thresholds (i.e., translational or rotational motion parameters less than 2 mm or 2°). We also calculated frame-wise displacement (FD), which indexes the volume-to-volume changes in head position. There were no significant group differences in mean FD (*t* = 0.601, *P* = 0.549) between patients with MDD (0.141 ± 0.066) and healthy controls (0.149 ± 0.073). Then, individual structural images were co-registered with the mean functional image. After the transformed structural images were removed of non-brain tissue, the individual whole brain masks were applied to individual functional images to extract the global signal, i.e., the BOLD signal time series averaged across all brain voxels. Finally, we calculated the variable coefficient (VC = standard deviation/mean) of the global signal for each subject. This measure reflects the relative amplitude of variation in the whole brain spontaneous neural activity during resting state (Wong et al. 2012, 2013, 2016) (Fig. 1).

### Statistical analysis

All statistical analyses were performed by using the SPSS19.0. A two-sample *t*-test was used to compare the VC of the global signal between patients with MDD and healthy controls. In patients with MDD, the association between the VC and the decline rate of HRSD scores was calculated by using Pearson correlation coefficient. According to the decline rate of HRSD scores, we further subdivided the patients into a non-responding depression group (NRD, N = 26, decline rate ≤ 50%) and a treatment-responsive depression group (TRD, N = 21, decline rate > 50%). A two-sample *t*-test was used to test the difference in VC between NRD and TRD groups. Then, receiver operator characteristic (ROC) curve analysis for VC was used to determine the cutoff value associated with optimal sensitivity and specificity for distinguishing TRD from NRD. In addition, univariate linear and logistic regression analyses in the patient group were used to assess the predictive value of the VC for the decline rate of HRSD scores and the antidepressant treatment response (NRD or TRD), respectively. For these analyses, two-tailed *P* < 05 was considered to indicate significance.

## Results

**Fig. 2:**
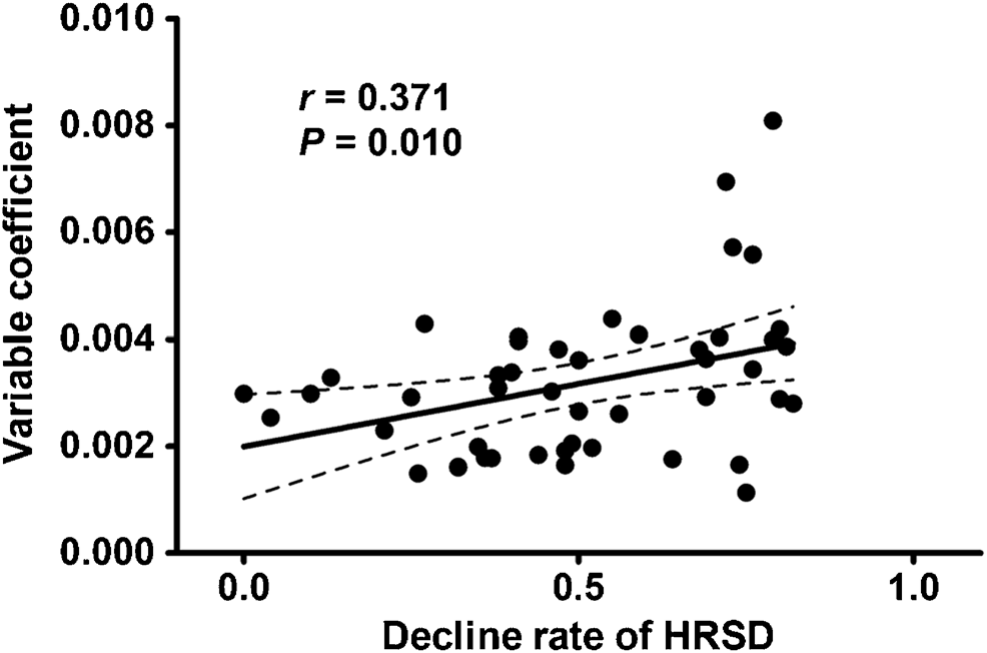
Correlation between the variable coefficient of the global signal and the decline rate of HRSD. Abbreviations: HDRS, Hamilton Depression Rating Scale

**Fig. 3:**
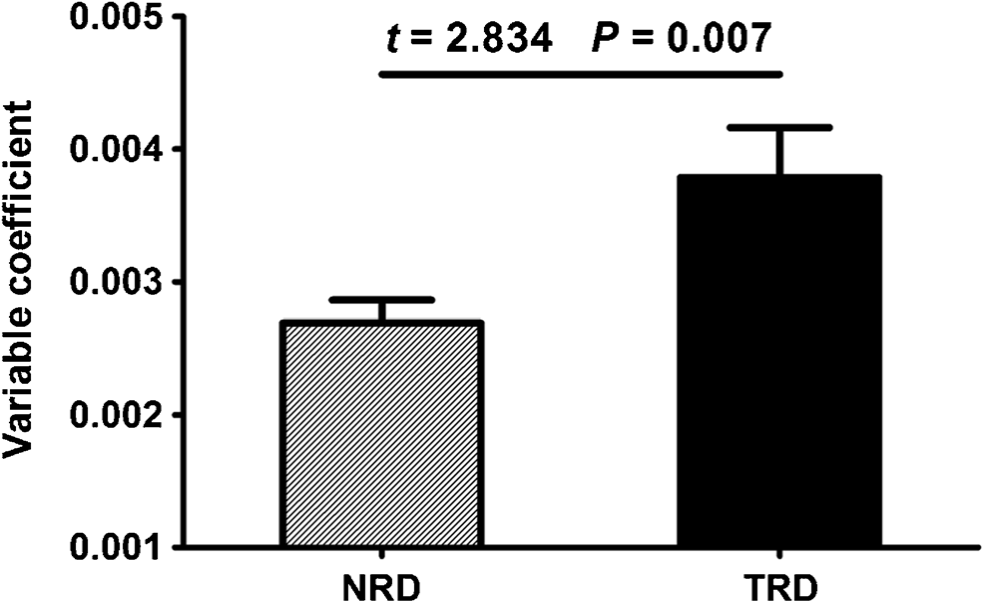
Difference in the variable coefficient of the global signal between the NRD and TRD groups. Abbreviations: NRD, non-responding depression; TRD, treatment-responsive depression

**Fig. 4:**
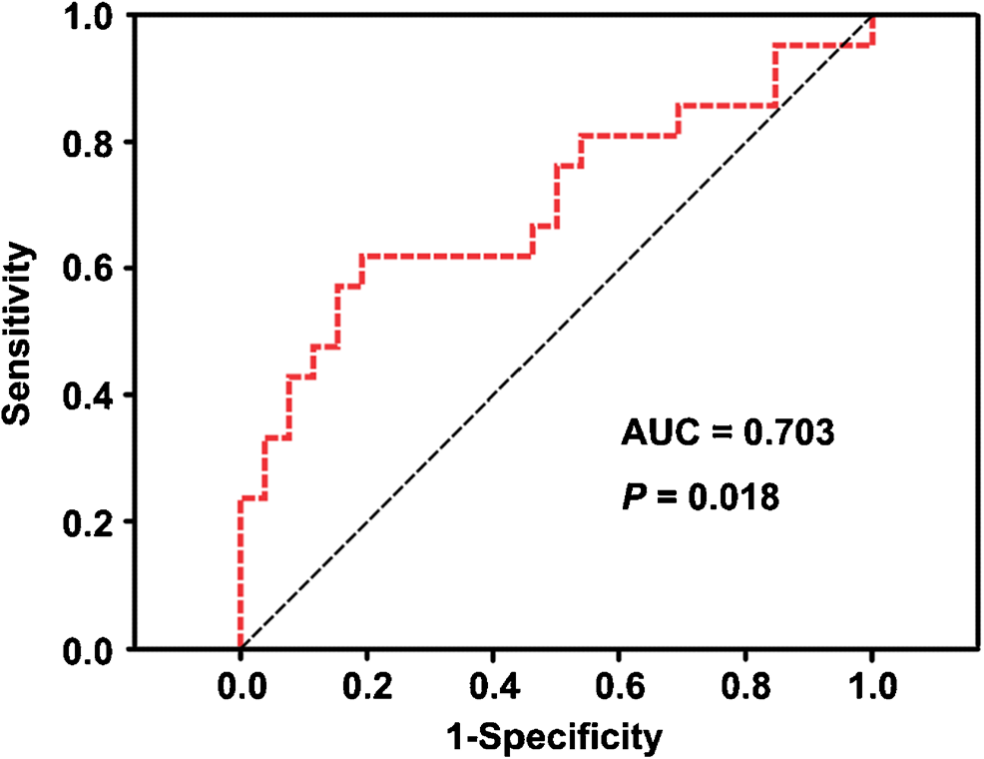
ROC curve using the VC to distinguish TRD from NRD. Abbreviations: AUC, area under the curve; NRD, non-responding depression; ROC, receiver operating characteristic; TRD, treatment-responsive depression; VC, variable coefficient

There was no difference (*t* = − 0.640, *P* = 0.524) in VC between patients with MDD (0.0030 ± 0.0012) and healthy controls (0.0032 ± 0.0014). However, we found a significant positive correlation (Pearson correlation coefficient *r* = 0.371, *P* = 0.010) between the VC and the decline rate of HRSD scores in the patients (Fig. 2). Compared with the NRD group (0.0027 ± 0.0009), the TRD group (0.0038 ± 0.0017) had a higher VC (*t* = 2.834, *P* = 0.007) (Fig. 3). The ROC analysis revealed that the area under the curve (AUC) of the VC was 0.703 (*P* = 0.018, 95% confidence interval = 0.546–0.861), indicating that the VC could be used to differentiate TRD from NRD (Fig. 4). At the optimal cutoff VC of 0.00342, the sensitivity and specificity were 0.619 and 0.808, respectively. In addition, the linear and logistic regression analyses showed that the VC was a significant predictor of the decline rate of HRSD scores (*β* = 58.519, *t* = 2.678, *P* = 0.010) and the antidepressant treatment response (odds ratio [OR], 2.034; 95% confidence interval [CI]: 1.136, 3.642; *P* = 0.017).

## Discussion

Based on resting-state fMRI data, we investigated the association between the global signal variance and short-term antidepressant response for the purpose of identifying an objective and easy-to-get marker to predict early treatment outcome of MDD. Despite no difference in the global signal variance between patients and controls, we found a significant positive correlation between the variance and the decline rate of HRSD scores in the patient group. Compared with the NRD, the TRD had a higher global signal variance. ROC analysis revealed that the global signal variance exhibited a good ability to differentiate TRD from NRD. In addition, linear and logistic regression analyses showed that the global signal variance was a significant predictor of the early therapeutic efficacy.

BOLD signal detected by fMRI is a complex measure that is influenced by cerebral blood flow (CBF), cerebral blood volume (CBV) and cerebral metabolic rate of oxygen (CMRO2) (Buxton et al. 2004). Changes in resting-state BOLD signal may result from any factor that affects the interaction of the 3 physiological parameters. These factors also include multiple potential confounds arising from head motion, cardiac and respiratory cycles, arterial CO_2_ concentration, blood pressure/cerebral autoregulation, and vasomotion (Murphy et al. 2013). As an integrated reflection of the BOLD signal, the global signal is found to share variance with non-neuronal noise, such as head motion, hardware artifacts, low-frequency respiratory volume and cardiac rate (Chang and Glover 2009; Power et al. 2014, 2016). Thus, various methods have been proposed to remove the global signal effects (Anderson et al. 2011; Chen et al. 2013; Macey et al. 2004). However, in recent years, a growing body of evidence suggests that the global signal also includes a neuronal component that may contribute to specific cognitive and clinical states. For instance, Yang et al. observed increased global signal variability in schizophrenia but not in bipolar disorder (Yang et al. 2014). Wong et al. found that ingestion of caffeine decreases global signal amplitude (Wong et al. 2012); the global signal amplitude exhibits a significant negative correlation with EEG vigilance measures across subjects in the eyes-closed condition (Wong et al. 2013); changes in the global signal amplitude between the eyes open and eyes closed states are related to changes in EEG vigilance (Wong et al. 2016). Chen et al. demonstrated that the global signal is strongly correlated with the default mode network components (Chen et al. 2012). Schoelvinck et al. reported that the spontaneous fluctuations in the local field potential measured from a single cortical site in monkeys show positive correlations with fMRI signals over nearly the entire cerebral cortex (Scholvinck et al. 2010). These findings suggest that the global signal, especially its variance, is tightly coupled to underlying neural activity that has biological significance. However, we found no difference in the global signal variance between patients with MDD and healthy controls in this study.

Previous studies have provided evidence that early drug response occurring within the first 2 weeks of ADM may predict eventual treatment outcome (Brannan et al. 2005; Henkel et al. 2009; Katz et al. 2004; Szegedi et al. 2003; van Calker et al. 2009). Many markers may serve as predictors of treatment response. For example, a prior study demonstrated that an increase in joy after 2 weeks of treatment is strongly specific for later antidepressant response and remission in MDD (Gorwood et al. 2015a). Gorwood et al. found that motivation is the most impaired in depressed patients, responds best to treatment and shows the best predictive value for antidepressant treatment response in the Multidimensional Assessment of Thymic States (MAThyS) rating scales (Gorwood et al. 2015b). Early improvement in depressive symptoms assessed by the Hamilton Rating Scale for Depression (Vermeiden et al. 2015) or the 16-item Quick Inventory of Depressive Symptomatology (Sakurai et al. 2013) can also predict eventual response. Beck et al. reported that repeated testing of the cortisol awakening response between baseline and after 10 days of treatment is able to predict antidepressant treatment outcome after 6 weeks of treatment (Beck et al. 2015). Furthermore, recent neuroimaging studies revealed that imbalanced interhemispheric functional coordination and impaired network topological architecture can be used for discrimination of TRD and NRD, suggesting they may be neural traits underlying the prediction of early therapeutic outcome in MDD (Hou et al. 2016a, b). Likewise, an objective and easy-to-get neuroimaging marker, i.e., variance of the global signal, was found to be able to effectively distinguish TRD from NRD in this study. This finding is of clinical value because this marker can help clinicians easily identify which patients will ultimately respond to treatment and decide at an earlier stage to continue or change treatment, thereby preventing delay, increasing treatment compliance, and decreasing morbidity.

There are several limitations to the present study that should be noted. First, consistent with many previous studies on ADM effectiveness and prediction (Hou et al. 2016a, b; Korgaonkar et al. 2015; Li et al. 2013; Shen et al. 2015), different antidepressants were used in the current study, which may influence our interpretation. The heterogeneous drug regimens reflect the natural treatment course of MDD because medications were prescribed by treatment clinicians according to the physical status and depression severity of the patients. However, emerging evidence has demonstrated that different drugs may trigger antidepressant responses in different ways (Gideons et al. 2014). Thus, this study should be considered a pilot study. Further studies with homogeneous patients using the same antidepressant are expected in the future to test the reproducibility of the current findings. Second, due to the absence of a placebo control group, we are unable to draw a definite conclusion about the specificity of the association between variance of the global signal and antidepressant response. Third, this study did not collect MRI data after 2 weeks, so whether variance of the global signal is altered with antidepressant treatment remains unclear. In the future, a long-term follow-up study including MRI scans before and after treatment should be conducted to clarify this issue. Fourth, depression severity of the MDD patients was not assessed after treatment for more than 2 weeks due to some patients’ refusal to participate or hospital discharge. This may prevent us from drawing a more definite conclusion on the role of the global signal variance in the prediction of antidepressant medication outcomes. Finally, artifacts from cardiac and respiratory noise are prevalent in resting-state fMRI analyses (Murphy et al. 2013). Thus, an advisable pre-processing step is to remove physiological noise from the data using simultaneously collected pulse and respiration data. However, physiological data were not collected in this study.

In conclusion, this study found that variance of the global signal in resting-state fMRI data can be used to predict early antidepressant response in MDD. This finding may provide clinicians a useful approach to find an appropriate therapy for individuals with MDD at the earliest opportunity.
